# Feasibility, benefit and risk of systematic intraoperative cholangiogram in patients undergoing emergency cholecystectomy

**DOI:** 10.1371/journal.pone.0199147

**Published:** 2018-06-28

**Authors:** Pouya Iranmanesh, Olivier Tobler, Sandra De Sousa, Axel Andres, Jean-Louis Frossard, Philippe Morel, Christian Toso

**Affiliations:** 1 Department of Digestive and Transplant Surgery, Geneva University Hospitals and Faculty of Medicine, Geneva, Switzerland; 2 Hepato-Pancreato-Biliary Centre, Geneva University Hospitals and Faculty of Medicine, Geneva, Switzerland; 3 Department of Gastroenterology, Geneva University Hospitals and Faculty of Medicine, Geneva, Switzerland; Texas A&M University, UNITED STATES

## Abstract

**Background:**

The role of intraoperative cholangiogram (IOC) during cholecystectomy is debated. The aim of the present study was to evaluate the feasibility, benefit and risk of performing systematic IOC in patients undergoing cholecystectomy for acute gallstone-related disease.

**Methods:**

Between July 2013 and January 2015, all patients admitted for an acute gallstone-related condition and undergoing same-hospital-stay cholecystectomy were prospectively followed. IOC was systematically attempted and predictors of IOC failure were analyzed.

**Results:**

Among the 581 enrolled patients, IOC was deliberately not performed in 3 cases. IOC was successful in 509/578 patients (88.1%). The main predictors of IOC failure were age, body mass index, male gender and associated acute cholecystitis. Thirty-two patients with suspected common bile duct stone on IOC underwent 38 unnecessary negative postoperative common bile duct investigations (32/509, 6.3%). There was one IOC-related adverse outcome (mild pancreatitis, 1/578, 0.2%).

**Conclusions:**

IOC can be successfully and safely performed in the majority of patients undergoing cholecystectomy for acute gallstone-related disease. Although its positive predictive value is suboptimal and results in a number of unnecessary postoperative common bile duct investigations, IOC accurately rules out common bile duct stones in patients with acute gallstone-related conditions.

## Introduction

Acute gallstone-related disease represents a heavy burden in terms of financial cost, and number of emergency room visits, accounting for more than one million urgent medical consultations in the United States yearly [1,2]. This disease includes acute cholecystitis, gallstone migration into the common bile duct (CBD), acute cholangitis and gallstone pancreatitis. According to current guidelines, patients admitted with an acute gallstone-related disease should undergo laparoscopic cholecystectomy during the same hospital stay [3–5]. The role of intraoperative cholangiogram (IOC) during elective and emergency cholecystectomy is debated. Some authors advocate for its systematic use [6–8] and others advise for a selective use in patients with abnormal liver function tests (LFT) [9–11]. The guidelines of the American Society for Gastrointestinal Endoscopy (ASGE) and the Society of American Gastrointestinal and Endoscopic Surgeons (SAGES) make the use of IOC dependent on the institutional strategy and patients’ individual risk of presenting a CBD stone [12–14]. IOC allows for the identification of CBD stones, the early detection of biliary lesions and, for some authors, a decreased readmission rate after cholecystectomy [7,8,15,16]. Conversely, it also accounts for an increased operating room time (and costs), and carries a risk of adverse outcome such as biliary lesions [9–11]. Technical reasons such as severe inflammation or narrowness of the cystic duct can lead to IOC failure. No study in the current literature specifically focuses on the role of IOC during cholecystectomy for acute gallstone-related diseases. The objective of the present study was to analyze the feasibility, benefit and risk of performing systematic IOC in a cohort of patients undergoing cholecystectomy for acute gallstone-related disease.

## Materials and methods

### Setting, design and interventions

This study was a retrospective analysis of a prospective database created between July 2013 and January 2015 at the Geneva University Hospitals, Geneva, Switzerland. In this institution, IOC is systematically performed during all cholecystectomies. Patients presenting to the emergency room with an acute gallstone-related disease were classified according to the ASGE/SAGES guidelines [12] as low-, intermediate- and high-risk of presenting a CBD stone. These patients underwent laparoscopic cholecystectomy during the same hospital stay and IOC was systematically attempted during the surgical procedure. According to the institution guidelines based on a randomized controlled trial [17], high-risk patients (defined by bilirubin level > 4 mg/dL, acute cholangitis according to the revised Tokyo guidelines [18], CBD stone confirmed on radiologic imaging or gallstone pancreatitis according to the revised Atlanta classification [19]) were scheduled for a preoperative CBD assessment first. This assessment was performed by either endoscopic ultrasound (EUS) or magnetic resonance cholangiopancreatography (MRCP), followed, when necessary, by CBD clearance by endoscopic retrograde cholangiopancreatography (ERCP), and a subsequent cholecystectomy with IOC. Low-risk (normal LFT) and intermediate-risk patients (abnormal LFT without high-risk criteria) were planned for initial cholecystectomy with IOC.

All patients with a suspicion of CBD stone on IOC (positive IOC) were scheduled for a EUS or MRCP after surgery. All patients with no suspicion of CBD stone on IOC (negative IOC) were followed-up during a year after discharge to track readmissions and CBD investigations for missed CBD stones.

A classical four ports laparoscopic cholecystectomy was the standard procedure. After exposure and identification of the elements of the Calot triangle, a small transverse cut was performed in the cystic duct close to the gallbladder infundibulum with laparoscopic scissors. A 4-French cholangiogram catheter (Cook Medical, Bloomington, IN, USA) placed on an Olsen forceps (Karl Storz, Tuttlingen, Germany) was then inserted into the cystic duct. After verifying the absence of leakage at the catheter insertion site, contrast media (Accupaque™, GE Healthcare, Chicago, IL, USA,) diluted in NaCl 0.9% with a 1:1 ratio in a 20 ml syringe was injected under fluoroscopic vision (Ziehm Vision FD, Ziehm Imaging GmbH, Nuremberg, Germany), first with a low output to form a thin layer allowing better visualization of filling defects corresponding to CBD stones, and then with a high output to visualize the biliary tree and the duodenal passage.

### Inclusion criteria

All patients > 16 years presenting to the emergency room with a gallbladder stone and one of the following acute gallstone-related conditions were included:

- Intractable biliary colics, defined by sudden right upper quadrant (RUQ) pain lasting > 6 hours
- Acute cholecystitis, defined by clinical (fever, presence of Murphy sign) and ultrasound (gallbladder wall thickness >4 mm, striated gallbladder wall, perivesicular fluid) criteria
- Suspected CBD stone migration (sudden RUQ and/or epigastric pain, associated with elevated LFT)
- Acute cholangitis as defined by the 2013 revised Tokyo guidelines [18]
- Gallstone pancreatitis as defined by the revised Atlanta classification [19]

All patients were planned for laparoscopic cholecystectomy with IOC during the same hospital stay.

### Exclusion criteria

Patients with following criteria were excluded:

- Indications for a delayed cholecystectomy (including acute cholecystitis with symptoms duration > 7 days, or decision to differ surgery in view of preoperative assessment in high-risk patients)
- Previous cholecystectomy
- Associated liver, bile duct or pancreas neoplasia
- Patient or legal caretaker refusal to undergo cholecystectomy

### Collected data

Demographic characteristics such as gender, age and body mass index (BMI) were collected. Clinical data included number of patients with associated acute cholecystitis, gallstone pancreatitis and fever (tympanic temperature ≥ 38.3°C) on admission. Primary outcome was number of successful IOC. Secondary outcomes were number and reasons of IOC failure, number of CBD stones discovered on IOC, number of CBD stones missed on IOC, number of unnecessary CBD endoscopic or radiologic procedures performed due to false positive IOC and number and types of complications linked to CBD investigations (including IOC, EUS, MRCP and ERCP) according to the Dindo-Clavien classification of surgical complications [20]. Post-discharge readmissions and CBD investigations were tracked for all patients during one year.

### Statistical analysis

All data were analyzed using SPSS Statistics version 24.0 (IBM Corporation, Armonk, NY, USA). Primary and secondary outcomes were compared among groups using Mann-Whitney and Fisher’s exact test accordingly. Demographic and clinical predictors of IOC failure with a p-value <0.1 on univariate analysis were subsequently entered into a multivariate analysis based on a logistical regression. Sensitivity, specificity, positive and negative predictive values of IOC were calculated overall and for each three categories of CBD stone risk. The accuracy of IOC in detecting CBD stones was analyzed overall and for each three categories of CBD stone risk using a Chi-square test. A p-value ≤ 0.05 was considered significant.

### Ethical considerations

The study was approved by the institutional review board (NAC 12-050R). Informed consent was obtained for all patients. All data were anonymized before analysis.

## Results

**Fig 1:**
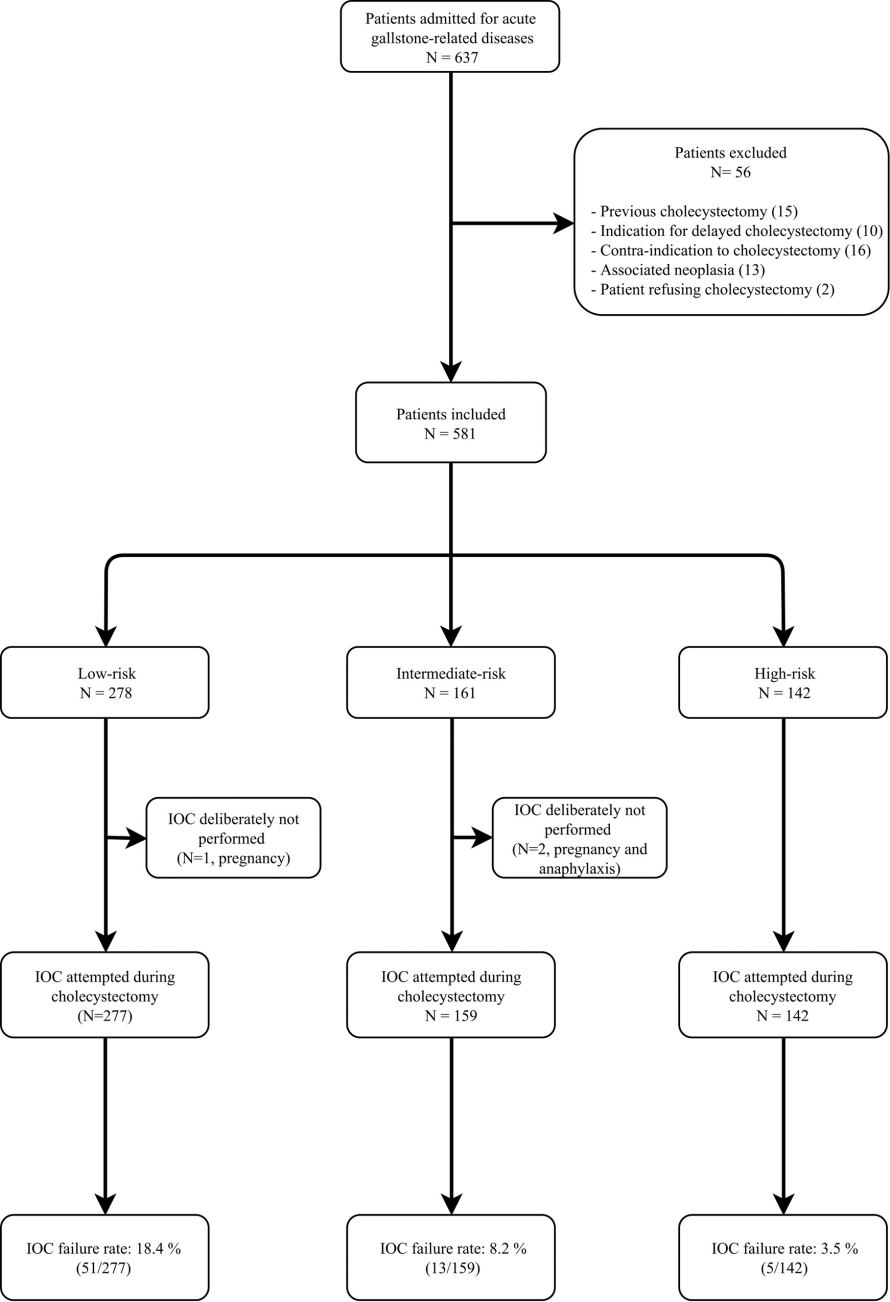
Flow-chart. IOC = intraoperative cholangiogram.

Eligibility was assessed for 637 patients during the study period and fifty-six patients were excluded according to the previously mentioned criteria (Fig 1) All patients underwent laparoscopic cholecystectomy during the same hospital stay. IOC was deliberately not performed in 3/581 patients (0.5%) due to pregnancy (N = 2) and previous severe anaphylaxis to contrast media (N = 1) respectively. IOC was attempted in all other patients (N = 578).

Age, BMI and gender ratio were similar between all groups. Fever was present in 61/581 patients (10.5%) without statistical difference between groups. There were significantly more patients with acute cholecystitis in the low-risk group (86.3% vs respectively 55.3% and 42.3%, P<0.01). Overall, male patients had a significantly higher rate of associated acute cholecystitis (184/254 (72.4%) vs 205/327 (62.7%), P = 0.0163). In the high-risk group, 33 patients had associated gallstone pancreatitis (33/142, 23.2%) (Table 1).

**Table 1:** Baseline characteristics.

	Overall (N = 581)	Low-risk (N = 278)	Intermediate-risk (N = 161)	High-risk (N = 142)
**Age [mean (SD) ; years]**	55 (7)	54 (18)	53 (20)	61 (19)
**BMI [mean (SD) ; kg/m**^**2**^**]**	28 (7)	28 (7)	27 (7)	27 (6)
**Gender (F:M)**	1.3:1	1.3:1	1.3:1	1.3:1
**Patients with fever (%)**	61 (10.5%)	29 (10.4%)	14 (8.7%)	18 (12.7%)
Patients with associated acute cholecystitis (%)*	389 (67%)	240 (86%)	89 (55%)	60 (42%)
Patients with associated gallstone pancreatitis (%)**	33 (5.7%)	0	0	33 (23.3%)

*There were statistically more patients with acute cholecystitis in the low-risk group.

**All patients with associated gallstone pancreatitis were classified in the high-risk group.

Among the low-and intermediate-risk groups (N = 278 and 161 respectively, 439 together), 385 patients (271 low-risk and 114 intermediate-risk) underwent initial cholecystectomy with IOC according to institutional guidelines and 54 patients underwent preoperative CBD investigations, including 7 low-risk patients, who did not require ERCP, and 47 intermediate-risk patients. Among these 47 intermediate-risk patients, 12 patients underwent preoperative ERCP and 3 of them required a second postoperative ERCP due to remaining CBD stones (15 ERCP in total). Among the high-risk group (N = 142), 6 patients underwent initial cholecystectomy with IOC and 136 patients underwent preoperative CBD investigation, including 93 ERCP. Among these 136 patients, a second preoperative ERCP was necessary to achieve complete CBD clearance in 5 cases (5/136, 3.7%) and a second postoperative ERCP due to remaining CBD stones in 6 cases (6/136, 4.4%). Two patients with normal preoperative EUS had CBD stones identified on IOC and underwent postoperative ERCP (2/136, 1.5%). Overall, there were 5 ERCP failures out of a total of 165 ERCP (5/165, 3%), including 3 preoperative ERCP failures (no CBD stone was found on IOC for 2 patients and open CBD exploration was performed for the third patient) and 2 postoperative ERCP failures in 2 low-risk patients with normal preoperative liver function tests, who underwent percutaneous cholangiography.

Table 2 summarizes the primary and secondary outcomes.

**Table 2:** Primary and secondary outcomes.

	Overall	Low-risk	Intermediate-risk	High-risk	Statistical comparison between the three groups (Fisher’s exact test)
	(N = 581)	(N = 278)	(N = 161)	(N = 142)	
**Number of deliberately unperformed IOC**	3/581 (0.5%)	1/278 (0.4%)	2/161 (1.2%)	0	Not significant
**Number of successful IOC**	509/578 (88.1%)	226/277 (81.6%)	146/159 (91.8%)	137/142 (96.5%)	Significant difference between low-risk and other groups (P = 0.03 and < 0.01)
**Number of failed IOC**	69/578 (11.9%)	51/277 (18.4%)	13/159 (8.2%)	5/142 (3.5%)	
**Number of IOC failure due to inflammation (acute cholecystitis)**	61/69 (88.4%)	48/51 (94.1%)	10/13 (76.9%)	3/5 (60%)	Not significant
**Number of IOC failure due to narrow cystic duct**	8/69 (11.6%)	3/51 (5.9%)	3/13 (23.1%)	2/5 (40%)	Not significant
**Patients with confirmed CBD stone**	130/581 (22.4%)	14/278 (5%)	34/161 (21.1%)	82/142 (57.7%)	Significant difference between all groups (P < 0.01)
**Patients with CBD stone initially discovered on IOC (%)**	38/509 (7.4%)	14/226 (6.2%)	22/146 (15.1%)	2/137 (1.5%)	Significant difference between all groups (P < 0.01)
**Patients with CBD stone initially discovered on CBD investigations (%)**	92/578 (15.9%)	0	12/159 (7.5%)	80/142 (56.3%)	
**Patients with CBD stone on IOC despite preoperative CBD clearance (%)**	7/107 (6.5%)	0	1/18 (5.6%)	6/89 (6.7%)	Not significant
**Patients with CBD stone missed on IOC (%)**	2/509 (0.4%)	0	2/146 (1.4%)	0	Not significant
**Patients with positive IOC but no CBD stone found postoperatively (%)**	32/48 (66.7%)	10/14 (71.4%)	13/20 (65%)	9/14 (64.3%)	Not significant
**Number of negative CBD investigations performed after positive IOC, including:**	38	11	17	10	Significant difference between low- and intermediate-risk groups (P < 0.01)
**- EUS**	23	7	12	4	
**- ERCP**	8	1	3	4	
**- MRCP**	7	3	2	2	

In 38/578 patients (6.6%), CBD stones were initially discovered on IOC, including 2/578 patients (0.3%) with normal preoperative CBD investigations (EUS in both cases) and 36/578 patients (6.2%) who underwent initial cholecystectomy. CBD stones were missed on IOC in 2/578 patients (0.3%), who underwent postoperative EUS due to persistently elevated LFT. In 7/107 patients (6.5%) who underwent preoperative ERCP clearance remaining CBD stones were discovered on IOC and confirmed on postoperative CBD investigations (1 MRCP and 8 ERCP). In 32/48 patients (66.7%), CBD stones were not confirmed postoperatively following positive IOC. These 32 patients underwent 38 negative postoperative CBD investigations overall, including 23 EUS, 7 MRCP and 8 ERCP. There were no complications linked to these 38 procedures. One high-risk patient who underwent preoperative ERCP with stone extraction and placement of a plastic CBD drain was readmitted electively 4 months later for ERCP and plastic drain removal; a remaining CBD stone was found and removed during the procedure. One intermediate-risk patient who underwent postoperative ERCP with stone extraction and placement of a plastic CBD drain was readmitted electively 3 months later for ERCP and plastic drain removal. There were no other readmissions and no other CBD investigations performed after discharge. There was one IOC-related complication (1/578, 0.2%), namely a mild pancreatitis, treated with supportive treatment (grade II according to the Dindo-Clavien classification [20]). The overall ERCP complication rate was 9/165 (5.5%), respectively 4/165 (2.4%) when considering severe complications only (grade ≥ III). There were 4 complications linked to preoperative ERCP, namely 3 cases of mild acute pancreatitis treated with supportive treatment (grade II) and 1 papillary bleeding treated with endoscopic hemostasis (grade IIIb). There were 5 complications linked to postoperative ERCP, namely 2 cases of mild acute pancreatitis treated with supportive treatment (grade II), 1 papillary bleeding treated with endoscopic hemostasis (grade IIIb), 1 early papillary stenosis treated with endoscopic papillotomy (grade IIIb) and 1 necrotizing acute pancreatitis requiring ICU admission (grade IV). All ERCP-related complications occurred in patients with confirmed CBD stones who required endoscopic extraction. Complications and readmissions for each group can be seen in Table 3.

**Table 3:** Complications and readmissions.

	Overall	Low-risk	Intermediate-risk	High-risk
	(N = 581)	(N = 278)	(N = 161)	(N = 142)
**Complications linked to IOC**				
**Mild pancreatitis (II)**	1	0	1	0
**Complications linked to ERCP**				
**Mild pancreatitis (II)**	5	0	3	2
**Papillary bleeding (IIIb)**	2	0	1	1
**Papillary stenosis (IIIb)**	1	0	1	0
**Severe pancreatitis (IV)**	1	0	1	0
**Readmissions (unplanned)**	0	0	0	0
**Readmissions (planned)**				
**CBD plastic stent removal**	2	0	1	1

The Roman numerals indicate the complication grade according to Dindo et al [20].

On the univariate analysis, age, male gender, associated acute cholecystitis and low risk of CBD stone were predictors of IOC failure (Table 4). BMI did not reach significance as a predictor of IOC failure (P = 0.066). Fever on admission was not related to a higher rate of IOC failure. The multivariate analysis showed that age, male gender, associated acute cholecystitis, BMI and low risk of CBD stone were independent predictors of IOC failure (Table 5).

**Table 4:** Predictors of IOC failure (univariate analysis).

	Succesful IOC (N = 509)	Failed IOC (N = 69)	P-value
**Age [mean (SD)]**	54.5 (18.6)	61.1 (19.2)	0.006*
**BMI [mean (SD)]**	27.7 (5.3)	29.6 (7.5)	0.066*
**Gender (F:M)**	298:211	28:41	0.006**
**Acute cholecystitis (N)**	324	61	<0.001**
**Fever on admission (N)**	50	11	0.141**
**Low risk of CBD stone**	226	51	<0.001**

* = Mann-Whitney test,

** = Fisher’s exact test

**Table 5:** Multivariate analysis of predictors of IOC failure.

	Odds ratio	95% CI	P-value
**Age**	1.02	1.004–1.037	0.015
**BMI**	1.08	1.030–1.136	0.002
**Gender**	0.49	0.276–0.858	0.013
**Acute cholecystitis**	3.19	1.283–7.924	0.013
**Low risk of CBD stone**	0.44	0.211–0.698	<0.001

The sensitivity, specificity, positive and negative predictive values of IOC can be seen in Table 6. The accuracy of IOC in detecting CBD stone was statistically significant overall and for each three categories of CBD stone risk (P < 0.0001).

**Table 6:** Sensitivity, specificity, positive and negative predictive values of IOC in detecting CBD stones.

	Overall	Low-risk	Intermediate-risk	High-risk
	(N = 581)	(N = 278)	(N = 161)	(N = 142)
**Sensitivity**	95.45%	100%	90.48%	100%
**Specificity**	93.10%	95.28%	89.60%	92.91%
**Positive predictive value**	56.76%	58.33%	59.40%	50%
**Negative predictive value**	99.54%	100%	98.25%	100%

## Discussion

The present results show that an IOC is feasible in most patients (509/578, 88.1%) undergoing cholecystectomy for acute gallstone-related conditions, when there are no contra-indications to IOC such as pregnancy or previous severe anaphylaxis to contrast media. IOC failure occurred more frequently in the low-risk group and was linked in nearly 90% of patients to an excessive local inflammation and an associated acute cholecystitis. There was only one minor adverse outcome (grade II) presumably linked to IOC (1/509, 0.2%).

Based on a multivariate analysis, acute cholecystitis, age, BMI,male gender and low risk of CBD stone were independent predictors of IOC failure. The relation between acute cholecystitis and IOC failure was linked to the presence of an inflammation extending to the porta hepatis. The very high prevalence of associated acute cholecystitis among low-risk patients (86%) explains the higher rate of IOC failure in this subgroup. The impact of age may be linked to a more delayed diagnosis in this category of patients, and the presence of more inflammation. A higher BMI leads to a more difficult exposure, as does male gender with the presence of more intra-abdominal fat [21].

The feasibility of IOC and its potential benefits in identifying CBD stones during cholecystectomy varied between the three ASGE/SAGES risk categories:

### Low-risk patients

Patients with isolated acute cholecystitis usually do not have elevated LFT and are classified as low-risk. This explains the significantly higher prevalence of acute cholecystitis (240/278, 86.3%, P<0.01), as well as the higher IOC failure rate (51/277, 18.4%, P < 0.01) in this group. Low-risk patients usually do not undergo preoperative CBD investigations or IOC in most institutions. Our data showed however that 5% (14/278) of these patients with normal preoperative LFT had a CBD stone, all of which were identified on IOC (no false negative). On the other hand, no CBD stones were found postoperatively in 10 patients with a positive IOC (10/226, 4.4%), resulting in 11 unnecessary CBD investigations, including one ERCP. This can be explained either by an incorrect reading of the IOC (false positive) or spontaneous postoperative gallstone migration into the duodenum. Thus, although these patients have the lowest probability of presenting a CBD stone and the highest rate of IOC failure, IOC during emergency cholecystectomy has a high sensitivity for detecting CBD stones in this population, but at the cost of negative postoperative CBD investigations.

### Intermediate-risk patients

Among the 114 patients who underwent initial cholecystectomy in this group, the vast majority of CBD stones (22/24, 91.7%) were discovered on IOC. IOC missed CBD stones in the 2 other patients, who underwent postoperative CBD investigations due to persistently elevated postoperative LFT. Forty-seven patients underwent preoperative CBD investigations, including 15 preoperative ERCP procedures. There was one preoperative ERCP failure in a patient who subsequently did not have any CBD stone on IOC. Interestingly, one patient with normal preoperative EUS had a CBD stone found on IOC, which was removed by postoperative ERCP. Although this might be due to a false negative result on EUS, intraoperative gallstone migration due to cystic duct manipulations or spontaneous interval migration are alternative explanations [22]. In this group, no CBD stones were found postoperatively in 13 patients with positive IOC (13/146, 8.9%), leading to 17 unnecessary CBD investigations, including 3 ERCP. As mentioned in the ASGE/SAGES guidelines [12], an initial cholecystectomy strategy among intermediate-risk patients mandates the use of intraoperative biliary imaging such as IOC. If a strategy with preoperative CBD investigation and clearance is selected, IOC still remains useful due to the possibility of spontaneous or iatrogenic gallstone migration in the CBD.

### High-risk patients

The ASGE/SAGES guidelines [12] recommend systematic preoperative CBD investigations and clearance for these patients. Preoperative ERCP was successfully performed in 80 out of 82 patients with CBD stone. One patient with ERCP failure underwent open CBD exploration and the other did not have a CBD stone on IOC. Eight patients (8/142, 5.6%), including 6 patients with preoperative ERCP clearance and 2 with normal preoperative EUS, required additional postoperative ERCP clearance due to CBD stones seen on IOC. Nine patients with positive IOC did not have CBD stones, resulting in 10 unnecessary postoperative procedures, including 4 ERCP. Although few studies analyzed the risk of interval migration in patients with CBD stones, it is possible that patient at high-risk of choledocholithiasis may have a higher risk of spontaneous interval migration, potentially linked to the presence of numerous small stones in the gallbladder or a larger cystic duct. Thus, the risk of interval migration (approximately 12% according to Frossard and al. [22]) and incomplete preoperative CBD clearance (6/142, 4.2% in this study), together with a very low rate of IOC failure in this group (5/142, 3.5%), justify performing systematic IOC in these patients as well.

There were more negative postoperative CBD investigations performed in the intermediate-risk group compared to the 2 other groups. Since intermediate-risk patients undergo initial cholecystectomy despite elevated LFT at our institution, surgeons may show a higher level of suspicion for CBD stones and more easy use of CBD investigations after surgery in the presence of a suspicious IOC image. This is probably less the case in low- or high-risk patients due to normal LFT and preoperative CBD clearance respectively.

Of note, out of the 38 negative CBD investigations performed following positive IOC, only 8 were invasive (ERCP). The remaining 30 procedures were either minimally (EUS, N = 23) or non-invasive (MRCP, N = 7).

Optimal quality of IOC images is critical for interpretation and might improve accuracy. Special care should therefore be given to ensuring the absence of leakage at the catheter insertion site by making an appropriately small cut on the cystic duct and correctly positioning the forceps. Presence of bones or other confounding elements in the fluoroscopic beam on the regions of interest should be avoided. Contrast media, even when diluted, quickly fills up the CBD and can result in saturated IOC images on which CBD stones might be difficult to identify: this can be prevented by using a thin layer of contrast media first. Air bubbles in the contrast media can mimic CBD stones and should be meticulously eliminated before injection.

Even though IOC exposes the patient to ionizing rays, the amount of radiation is minimal, ranging from 0.01 to 0.35 mSv and resulting in a lifetime risk of developing a new cancer of less than 0.001% [23]. Surgeons experience in performing IOC most likely diminishes patients exposure. Technical advances will probably make IOC even safer in the future by allowing fluoroscopic devices to produce constantly higher quality images with less radiation.

Although IOC increases overall operating room time, it can be performed quickly and accurately by a trained surgeon [24]. At our institution, IOC is therefore also performed for didactic reasons and takes less than 10 minutes most of the time. The material cost per procedure (contrast media and disposable catheter) is approximately 25 US dollars. The mobile fluoroscopic C-arm device is readily available in most operating rooms and does not increase the costs.

Although this was not the primary focus of our study, our data show that the accuracy of IOC is very good, with overall and categorized sensitivities and specificities of >90%. EUS and MRCP show similar accuracy in the literature, with respective sensitivities of 91–99% and 80–93%, and specificities of 83–94% and 87–96% [25–27]. Even though the positive predictive value of IOC in our study is only around 50%, resulting in a number of unnecessary postoperative procedures (a total of 38 for 578 patients), its negative predictive value is nearly 100%, making it an excellent, safe and inexpensive investigation to exclude CBD stones.

This study has some limitations. The retrospective design and the fact that IOC is performed in all cholecystectomies in our institution did not allow us to include a comparison cohort (i.e. a group without IOC performed during cholecystectomy), although such a cohort would not have influenced the primary endpoint of the study (number of successful IOC). A blind analysis of all IOC images by the investigators was lacking, but these images were not available for all patients, while only key images (as opposed to the full dynamic sequence) were saved for the others. Finally, the surgical procedures were performed by surgeons with different levels of experience, potentially leading to a bias in the failure rate of IOC.

In conclusion, our results show that IOC is feasible in the majority of patients who undergo emergency cholecystectomy, with a higher failure rate among patients with associated acute cholecystitis. The suboptimal positive predictive value of IOC results in a modest increase in the number of negative postoperative CBD investigations, most of which are non-invasive (EUS or MRCP). Despite this fact, performing IOC is a good, safe and inexpensive way to identify or rule out CBD stones in patients with acute gallstone-related disease at all levels of CBD stone risk.

## Supporting information

S1 Dataset Table of participants data used in the present study. (XLSX)
